# Reviews and Book Notices

**Published:** 1882-08

**Authors:** 


### Reviews and Book Notices.

ARTICLE XV.—SENSATION AND PAIN. By CHARLES FAYETTE TAYLOR, M.D. A Lecture delivered before the New York Academy of Sciences, March 21, 1881, being one of the Public Course for 1880–81. 12mo, pp. 77, cloth, 75 cents. New York: G. P. Putnam's Sons, 27 and 29 West 23d St. 1881. Chicago: Jansen, McClurg & Co.

This is one of those rare gems of literature, which could not have been written before the latter half of this century. The terminal statement made, that Carpenter, Bain, Spencer, Bastian, Maudsley, Tuke, Huxley, etc., had been consulted before the author prepared his lecture, was not intended for the critics, who could well enough imagine that, yet the subject is treated in an original as well as masterly manner, and illustrated with a variety of charming anecdotes, which render the book just as interesting to a schoolboy as to a metaphysician.

After giving a short description of the function of the nervous system, a condensation, as it were, of the first part of Herbert Spencer's Psychology, Dr. Taylor explains the relation between consciousness, sensation and pain, explains illusions of some sorts, and gives a mode of performing minor operations without pain, which physicians of experience have all practiced more or less. A perusal of this little book will well repay every practitioner, but its reading is fully as interesting to those who have no practical knowledge of medicine. It will be remembered Dr. Taylor is free from all the prejudices of the age, and astonished the medical profession of New York not long ago, by pointing out the ill results of disuse of the sexual organs among the unmarried women of the higher class. In the book under consideration, the same principle of looking after truth, whatever the consequences may be, has been followed throughout.

ARTICLE XVI.—LECTURES ON ELECTRICITY (DYNAMIC AND FRANKLINIC), IN ITS RELATIONS TO MEDICINE AND SURGERY. By A. D. ROCKWELL, A.M., M.D., Electro-Therapeutist to the New York State Woman's Hospital, etc. 8vo, cloth, pp. 122. Profusely Illustrated. New York: William Wood & Co. 1881.

For those who possess the larger work of the author and Dr. Beard, this will serve the purpose of a valuable supplement; yet this little book is complete in itself, and suffices for the ordinary wants of those physicians who have occasion to use electricity once in a while.

The issue of this little book is timely, to say the least, for since Dr. Poole, of Ontario, propounded his theory that electricity was nothing but a paralyzer, and that Dr. S. V. Clevenger said that the medical applications of electricity were few, and gave the name of quacks to some of its most prominent advocates in this country, the sale of batteries must have suffered a reverse. Dr. Rockwell's little book will strengthen the faith of the many, and is surely worth reading by even those who know so little about batteries as to suppose that the results depend on the physician's judgment, instead of on the different currents used.

ARTICLE XVII.—"DOCTOR, WHAT SHALL I EAT?" A Handbook of Diet in Disease, for the Profession and the People. By CH. GATCHELL, M.D., formerly Professor of Theory and Practice, University of Michigan. 12mo, cloth, pp. 147. Published by the Author. Milwaukee: 1880.

The author recommends beef, mutton, chicken, eggs and milk, and discards pork, veal, salt meats, fish and cheese. He recommends to substitute olive for cod-liver oil (?) when the latter disagrees. Wheat flour and rice thoroughly cooked are valuable.

Here is the rule given in regard to fever-patients: Give no solid food to a fever-patient; let the food be simple, but nutritions; give it at frequent intervals, and in small quantities; let the patient have all the cold water he wants to drink. Follow the recipes of various articles of diet. This is the plan followed throughout, and it is so thorough as to satisfy most readers, patients and cooks.

This work is constructed on the principle of the manuals which have been flooding the market lately; compendia from larger works. That the condensation has been well done, there is no doubt, and the author had the good sense to leave out every patent article of diet or artificial food.

On the whole, Dr. Gatchell has produced a useful little book, which should be found in every household.

ARTICLE XVIII.—A MANUAL OF ORGANIC MATERIA MEDICA, Being a Guide to Materia Medica of the Vegetable and Animal Kingdoms, for the use of Students, Druggists, Pharmacists and Physicians. By JOHN M. MAISCH, PHAR. D., Professor of Materia Medica and Botany in the Philadelphia College of Pharmacy. Philadelphia: Henry C. Lea & Co., 1882.

A book evidently written for a purpose, and not simply for the purpose of writing a book. It is comprehensive, inasmuch as it refers to all, or nearly all, that is of essential value in organic materia medica; clear and simple in its style; concise, since it would be difficult to find in it a superfluous word, and yet sufficiently explicit to satisfy the most critical.

The system of classification of subject matter is natural and obvious, the first step being their separation into animal and vegetable substances. The first of these grand divisions is subdivided into, first, that in which the entire animal is used, e.g., the leech, coccus, cantharis, etc.; next, animal products, e.g., eggs; next, animal tissues, as sponge, gelatin, etc., and, lastly, secretions and excretions, as musk, castor, etc.

A similar method of classification applied to vegetable matters permits their separation into cellular and non-cellular structures, and the subdivision of these classes into roots, rhizomes, tubers, bulbs, twigs, woods, barks, leaves, leaflets, herbs, flowers, petals, fruits, seeds, etc., and next into sugars, extracts, gums, resins, oils, balsams, etc.

The text is freely illustrated with wood-cuts, which cannot fail to be valuable in familiarizing students with the physical, microscopic and macroscopic appearance of drugs.

The work is preceded by a table of contents, and completed with that, without which no book should be considered complete, *i. e.*, an index.

In fact, the little book is just what it pretends to be, and as such is worthy of unqualified commendation. W. H.

ARTICLE XIX.—A MANUAL OF MIDWIFERY. By ALFRED MEADOWS, M.D., London, F.R.C.P., Assisted by ALBERT J. VENN, M.D., M.R.C.P. Fourth Edition, Revised and Enlarged, with 137 Illustrations. 12mo, cloth, pp. 498. G. P. Putnam's Sons. New York: 1882.

This book is in brevier type, a common thing with the text-books and periodicals of Great Britain, which should not, however, be encouraged, for the impairment of sight resulting from the reading of small type more than counterbalances the saving of money. Needless to mention that the largest part of this book is formed of condensations, yet it also contains some statcments not to be found in some larger works. The class of people for whom this book will be most useful are the young physicians who need to take an obstetrical book along when called to cases of labor, as this one is not at all bulky. But while we have Ramsbotham, Playfair, Lusk, and so many other valuable text-books, we do not see the advantage of shorter works. However, this manual is not by any means to be classed with the cramming manuals, for it certainly contains more than the essentials of what it treats, and will confer all the necessary knowledge on those who make a careful study of it.

ARTICLE XX.—HOW WE FED THE BABY, TO MAKE HER HEALTHY AND HAPPY, WITH HEALTH HINTS. By C. E. PAGE, M.D. New York: Fowler & Wells, 1881.

The old proverb says, "Out of the fullness of the heart the mouth speaketh."

It is very evident that the author's heart is full of his baby, fuller, perhaps, than his baby would be of food, upon three meals a day, which seems to be the hobby upon which he would ride into printer's ink and notoriety.

The book is a protest against over-feeding of infants, and while it contains much that is true, contains nothing that is new, or that has not been taught and practiced by intelligent physicians for a generation.

Exception to this criticism should, however, be made in favor of the author's scheme of feeding an infant but three times daily—which would seem to those who, unlike the author, have had more than one baby, a somewhat too vigorous protest against over-feeding.

The author's citations from "Mark Twain" are funny, but can scarcely be regarded as authoritative. In his list of authors for reference let him add to Mark Twain, "Holy Job," and read what that authority says about book-making, and ponder it well before he publishes another book (or baby). W. H.

ARTICLE XXI.—A TEXT-BOOK OF PHYSIOLOGY. By M. FOSTER, M.A., M.D., F.R.S. Second American from the Third and Revised English Edition, with Extensive Notes and Additions, by E. S. REICHERT, M.D. 259 Illustrations. 12mo, leather, tinted edge, pp. 987. Philadelphia: Henry C. Lea's Son & Co., 1881. Chicago: Jansen, McClurg & Co.

A more compact and scientific work on physiology has never been published, and we believe ourselves not to be mistaken in asserting that it has now been introduced into every medical college in which the English language is spoken. This work conforms to the latest researches into zoölogy and comparative anatomy, and takes into consideration the late discoveries in physiological chemistry, and the experiments in localization of Ferrier and others. The arrangement followed is such as to render the whole subject lucid and well connected in its various parts. However, the amount of information contained in these 900 and odd pages is immense, and requires a good deal of pondering before the student's mind can become familiar with it. This is an unavoidable fault of modern text books, because of the wonderful stride just made in the various sciences connected with animal life.

ARTICLE XXII.—MEDICAL HERESIES HISTORICALLY CONSIDERED. A Series of Critical Essays on the Origin and Evolution of Sectarian Medicine, Embracing a Special Sketch and Review of Homœopathy, Past and Present. By GONSALVO C. SMYTHE, A.M. M.D., Professor, etc., etc. Philadelphia: Presley Blakiston, 1880.

The "Series of Critical Essays on the Origin and Evolution of Sectarian Medicine," indicated in the above title, is comprised in ninety-five pages of this little volume, and might with greater propriety be entitled an epitome or abstract of the history of certain medical heresies. Although doubtless interesting to those who have read little of the history of medicine, or to whom a wide range of medical literature is inaccessible, this narrative is inaptly termed a "series of essays," and still more inaptly entitled "critical."

The only heresy which the author has really essayed to criticise being that last and most stupendous of all, homœopathy. The zeal with which he assails this modern delusion impresses the reader that this is really his objective point of attack, the *raison d' etre* of the book, and that the preliminary narrative is merely a sort of skirmish before the battle.

While there can be no question of the justice of the author's criticisms, their wisdom may well be doubted. The time and labor wasted in combating heresies, whether theological, philosophical or medical, would be much more profitably utilized in the observation of facts and phenomena, the deduction of their underlying principles and the laws to which they are subordinated. Science, like philosophy, can afford to let heresies alone and pass them by unnoticed. All heresies must sooner or later perish, since they contain the elements of their own destruction, *i. e.*, the principle of negation. Upon such foundation no permanent superstructure can be reared; any edifice so constructed must depend for its maintenance upon factitious aid, and this is always largely composed of that sort of public sentiment which rallies at the cry of "persecution." "The blood of the martyrs is the seed of the church," and medical, like all other heretics, love to play the role of martyrs, and thrive upon "persecution."

While we deprecate all sectarian polemics in medicine, it must at the same time be said that if anyone is still unsatisfied of the absurdity of homœopathy, and has the time to waste in informing himself thereupon, he will find it sufficiently demonstrated in this little book to satisfy the most incredulous. W. H.

ARTICLE XXIII.—DRUGS THAT ENSLAVE. THE OPIUM, MORPHINE, CHLORAL AND HASCHISCH HABITS. BY H. H. KANE. Presley Blakiston, Philadelphia, 1881.

The author's object in the publication of this little book is to direct attention more forcibly to the evil effects of the opium habit, more especially, and also to those resulting from chloral and haschisch. He indicates, also, the danger incurred by medical men of establishing these habits in susceptible patients, by the incautious administration of the drugs under circumstances which seem to demand their use.

He suggests as a very efficient cause of much of the opium eating in the higher classes of society, the pernicious influence exercised by the examples of Coleridge, De Quincey and other opium slaves who have acquired eminence in the domain of belles-lettres, and very justly charges De Quincey with having "left a large debit on the side of truth, and handed down to succeeding generations a mass of ingenious lies * * *;" lies which have fascinated many a weak-minded victim, and lured him to his ruin. Of these the author cites examples, and we could add to the number.

The author regards with especial apprehension the dangers surrounding the hypodermic administration of these drugs, calls attention to the rapid dissemination of this method among the laity, and points out certain accidents likely to accompany subcutaneous medication, even when applied by experts. Among these he cites, from the experience of numerous observers, the occurrence of almost fatal syncope.

In announcing his ideas concerning the treatment of the opium habit, the author especially condemns the sudden withdrawal of the drug, and approves the gradual diminution of the dose as more humane, as well as more efficient. Reason, observation, and personal experience induce us to differ from the author's views upon this subject. If certain deleterious effects ordinarily follow the use of any drug whatever, and so surely that the drug may reasonably be indicated as their cause, whether the drug be opium, chloral, haschisch, or alcohol, its absolute withdrawal would seem to be the most rational and essential first step toward accomplishing the removal of the effects. Long continued observation will satisfy the majority that the "tapering off" process, will more frequently result in disappointment than in success, and the personal experience of any confirmed morphine eater will enable him to distinguish no difference in the tortures resulting from the withdrawal of the half or of the whole of his accustomed dose.

Moreover, the author announces as barbarous the sudden withdrawal of the drug after the method of Lowenstein (several of whose cases he cites), which appears from the record to have occupied nine and eighteen days respectively, while for the application of the gradual (his own) method he insists upon the preliminary signing of a paper "submitting him (the patient) to the entire control of the physician for ten days," a very wise precaution, but inconsistent with his expressed preference for the gradual (?) method.

A little more attention to grammatical accuracy would have been commendable in a work addressed to professional readers; for example, on page 38 we are told that "alcoholic stimulants are used by some habitués to increase the effect of the morphia, which *it* does to a certain extent. They delude themselves into the belief that they will be enabled to thus reduce the amount of morphia used, but in the majority of cases this is a fallacy." And on page 83: "I think that the doctor is right, with reference to idiosyncrasy, but I think, also, that the method of administering the drug had much to do with it."

The book contains much that is worth knowing to those ignorant of its subject matter, and will serve to direct attention to the unwarranted abuse of a most valuable therapeutic agent.

In conclusion, the author takes occasion, very properly, to censure physicians who encourage these dangerous habits in their patients, druggists who pander to them for the sake of gain, charlatans who profess to cure them by the substitute of one narcotic for another, and the (so-called) "religious press," which advertises these rascals. W. H.

ARTICLE XXIV.—THE DIET CURE; An Essay on the Relations of Food and Drink to Health, Disease and Cure. By T. L. NICHOLS, M.D., Author of "How to Live on Sixpence a Day," "How to Cook," "Esoteric Anthropology," "Human Physiology, the Basis of Sanitary and Social Science." New York: M. L. Holbrook & Co.

A sort of digest of dietetic philosophy, the whole science of hygiene in a nut-shell. The author is not only an M.D., but a philosopher of the school of the stoics, and an enthusiast, who seems to have accepted the conclusions of the Arab in the story, who, when butter was declared by the merchant to be as sweet as honey, inferred that honey was better than butter, and when honey was declared to be as clear as water, concluded that water was the best of all. So our author appears desirous of excluding everything from his dietary system as impure, except air and water, and these only in exceptional cases, since they, too, are often impure, and "dried apples," "these," he admits, "are delicious"—"when cooked with sugar."

On page 9 he says: "Let us imagine man placed upon this planet, fresh from the hand of the Creator; what would he eat? Precisely that for which *he was adapted.* He would smell, pick, eat the strawberry, the plum, the peach, the apple, the orange, the banana, the fig, the date, the clusters of ripe grapes. To these he would add all kinds of sweet nuts, and for variety, or pressed by hunger, he would eat tender leaves, as of lettuce or cabbage, and roots, as turnips or onions."

The author does not tell us what his man would do during six or eight months, with six feet of frost in the ground. Evidently Col. Sellers' diet of raw turnips and water was not original. "There's millions in it."

One of the author's works was omitted from the title page, to be referred to in the text *i. e.*, "a baking powder," prepared with which even "white bread" may be eaten without danger.

W. H.

ARTICLE XXV.—THE INCIDENTAL EFFECTS OF DRUGS; A Pharmacological and Clinical Hand-book. By Dr. L. LEWIN, Assistant at the Pharmacological Institute of the University of Berlin. Translated by W. T. Alexander, M.D. New York: William Wood & Co., 1882.

In the author's preface occurs the following: "But in the therapeutic employment of certain drugs, deviations sometimes occur from this typical, and, as one may say, normal action, whose recognition and correct interpretation are not always easy."

These deviations from the normal action of drugs, the author has classified etiologically into those dependent on individual peculiarity, those attributable to temporal or local influences, and those resulting from the quality of the drug.

Under the first of these classes, *i. e.*, individual peculiarity, he includes those physiological susceptibilitics termed idiosyncracies, both organic and chemical, or chemico-vital. Under organic idiosyncrasies he classifies "variations in the influence of external agencies upon homologous tissues," and also "those manifested by similar and dissimilar organs in the same individual under the influence of external agencies."

Under chemical idiosyncrasies are classed those dependent on "the presence in the body, in excess, of chemical substances which render more soluble than usual the medicinal agents introduced, or which enter into combination with them, thus forming new and directly deleterious agents," and "pre-existing pathological changes in organs, or diseases of the regulatory apparatus."

Under the head of "Temporal and local influences" he indicates more especially what has been aptly termed the "epidemic constitution" and climatic influences.

The deviations dependent on the quality of drugs he attributes to the following causes: *i. e*, to poorness of quality of the crude drug, to deterioration by age, to adulteration, or to variation in quality resulting from different modes of preparation.

In the book before us the author has elaborately discussed the anomalous effects and their various causes, in their relations to more than one hundred of the most valuable articles of the materia medica; he has enriched his treatise with citations from nearly four hundred authorities, and has accomplished his work in a style and manner which cannot fail to prove attractive to every student and scholar, for the evidences of laborious investigation, judicious classification and careful analysis, as well as for its clearness of expression, elegance of diction and conciseness of style.

This book will richly repay any reader for the time and attention bestowed upon its pages. The expert, if he finds nothing which has not already come within the range of his own observation, will, at least, find verifications of his experiences which will serve to corroborate them, while the tyro will learn the true interpretation of many perplexing problems, whose solution will surely puzzle his brain during the clinical experiences of his future professional life.

This book is a real contribution to medical science, a narrative of work done, for whose introduction to an English reading profession no small measure of commendation is due to the translator. The style of English into which the German has been rendered indicates that it is the work of one familiar not only with the German language, but also with his own, a qualification apparently regarded as superfluous by the majority of translators. The publisher has done his work in a manner befitting the excellence of the subject matter; the paper, type, composition and press-work are alike good, so that the book is not only "good reading," but "easy reading." W. H.

ARTICLE XXVI.—CONSPECTUS OF ORGANIC MATERIA MEDICA AND PHARMACAL BOTANY, Comprising the Vegetable and Animal Drugs; Their Physical Character, Geographical Origin, Classification, Constituents, Doses, Adulterations, Etc.—Table of the Tests and Solubilities of the Alkaloids Appended. By L. E. SAYRE, PH.G. Detroit: George S. Davis, Medical Book Publisher, 1880.

This will prove a valuable text-book for students, for whose use it purports to have been written. It is apparently the work of one practically familiar with his subject and competent to teach. It comprises a chart of botanic materia medica, in which the articles are tabulated under the captions: Natural order, officinal name, botanical name, common name, habitat, parts used, constituents, medical properties, dose and officinal preparations; chapters devoted to the geographical grouping of materia medica, to structural botany, to the botanical arrangement of plants, to the characteristics, constituents and adulteration of drugs, concluded with a very useful table of vegetable antidotes and incompatibles, and one of solubilities and tests of alkaloids.

Notwithstanding its syntaxical defects, the book is a useful contribution to medical literature, and is deserving of presentation in a better dress than that in which its publishers have invested it. The remarkable disproportion between its price and its mechanical demerits, will probably prevent a circulation to which its intrinsic merits entitle it. W. H.

ARTICLE XXVII.—FOOD FOR THE INVALID, THE CONVALESCENT, THE DYSPEPTIC AND THE GOUTY. By I. MILNER FOTHERGILL, M.D., Edin., Member of the Royal College of Physicians, London; Associate Fellow of the College of Physicians, Philadelphia, Etc., Etc.; and HORATIO C. WOOD, M.D., Professor of Materia Medica and Therapeutics, and Clinical Professor of Diseases of the Nervous System in the University of Pennsylvania; Member of the National Academy of Science. New York: MacMillan & Co., 1880.

The book consists of an introduction, which might be justly termed an essay on the philosophy of food, terse, concise, sententious, epigramatic almost, written by the free hand of a master, reminding one of some of the productions of the impressionist school of artists, by whom a single bold dash of color will give the entire life and character to the painting, and a collection of recipes for facilitating the preparation of wholesome and palatable food for the classes for whom they are intended.

The necessity for regulating the diet in accordance with constitutional requirements is indicated thus, "Taraxacum for the liver, and potash for the kidneys are all very well, but a correct and intelligently designed dietary is the main thing. When of old the doctor sagely shook his head and said: "Liver and kidneys," he was not the fool it has been recently the fashion to regard him. He saw through a glass darkly, but nevertheless correctly. He was in the dark, true; but daybreak was not far off. And again, "In most persons the system is not easily deranged, and excess is not swiftly followed by punishment, while in others the punishment follows quickly on the heels of the offense. These latter are quickly taught the relations of cause and effect; a rich meal means a bilious attack next day; a good dinner with sub-acid wines, a red hot great toe at no very distant period. But, sad to say, the voice of the avenging fate is only audible to a very fine ear, and is never heard by ordinary persons. They go on eating and drinking, guided, or rather led by their palate, which latter they whet with bitters. But in the far distance there is "gout poison" and "bile poison;" the danger signals are up, but they are not heeded until they have been passed, and then these individuals become wise after the event. It is a pleasant course they follow: why meet trouble half way? Events that are in the distance may not happen. Quite so! The feet of the avenging deities are shod with wool, etc. W. H.

ARTICLE XXVIII—A MEDICAL FORMULARY, Based on the United States and British Pharmacopœas, Together with Numerous French, German and Unofficinal Preparations. By LAWRENCE JOHNSON, A.M., M.D., Lecturer on Medical Botany, Medical Department of the University of the City of New York; Fellow of the New York Academy of Medicine, Etc. New York: William Wood & Co., 1881.

Everything should have what Gallic neighbors are wont to call a "raison d' etre," an excuse for its existence. If this book has such we fail to perceive it. It purports to "present in a manner convenient for ready reference the drugs and preparations in common use, etc.," thereby violating, at the same time, truth and syntax, and, "though based on the United States and British Pharmacopœas," does not include all the drugs and preparations contained in them, a number of the less important having been omitted." A modest confession, surely, concerning a book of less than four hundred pages, which presents very superficially what the works from which it is compiled treat more thoroughly. The metric system, which has been already so generally adopted, and which must soon be universally applied, is disposed of in a short paragraph in the introduction, and nowhere else in the whole work receives recognition.

It is simply one book more on the subject of pharmacy, which would be valuable if the others did not exist. Moreover, it assumes to be one of a "Library of standard medical authors," being in truth a compilation by a neophyte absolutely unknown in medical literature. W. H.

ARTICLE XXIX.—OPIUM SMOKING IN AMERICA AND CHINA. By H. H. KANE, M.D.

From every possible source Dr. Kane has collected the material for this interesting little volume. As an instance of the thorough investigation he has given the subject, he has hunted up the most incorrigible opium "fiends," giving them treatment and shelter free of charge, for the purpose of studying the habit. He has smoked himself many times, carefully noting the physiological phenomena. Every official likely to possess information he has written to or interviewed on the subject. The English opium trade with China receives pretty thorough ventilation, not to English credit. The amount of the opium trade, and its increase year by year, is tabulated. The growth of the habit in America is illustrated in startling detail. The author has concluded, however, that the habit can be thoroughly cured with ease, compared with the morphia or opium eating habit. New York: G. P. Putnam's Sons. W. L. D.

ARTICLE XXX.—THE PHYSICIAN HIMSELF, and What He Should Add to the Strictly Scientific. By D. W. CATHELL, M.D., late Professor of Pathology in the College of Physicians and Surgeons of Baltimore, etc.

This modest little volume, on a miscellaneous collection of subjects, which rarely find a place in literature, will be found a grateful companion by a large proportion of the medical profession. It consists of an essay on personal questions in medical practice, written in simple, colloquial style, in the form of a reply to a question, that the subjects may appear more direct and personal to the reader. There is not a subject connected with the personal welfare of the medical man mentally, morally or ethically, left untouched. The author says, in his first lines: "Professional tact and business sagacity are as necessary to the physician as the mariner's compass is to the navigator. There are gentlemen perfectly acquainted with the scientific aspects of medicine, who can tell you what to do for every ailment that afflicts humanity—but who, after earnest trial, have secured neither reputation or practice, because they lack professional tact and business sagacity; and there is nothing more pitiful than to see a worthy physician waiting year after year for practice that never comes." To such the author plainly says: "You will find that intellect, genius, temperance and other excellent qualities will all fail, unless you add ambition, self-reliance and agressiveness to them." To the young doctor he says: "You should, above all else, strive to start promptly on the road to success; for, unless you let the people know you are about, make some mark, and get a reputation in your first six or eight years, the probabilities are that you never will." Our old Mentor thoughtfully remembers the young Galen in his perplexity as to choice of location, and advises him as to many little things, even to the size and inscription on cards and signs, and details of office arrangements. The importance of cultivating methodical business habits is earnestly inculcated. The personal conduct of the physician under all circumstances is thoughtfully and sensibly advised upon. There is a convenient index appended, in which the various exigencies of professional life are alphabetically arranged and paged.

Every physician who has not reached the calm grays of age will find something of profit to himself in this little brochure, which so pleasantly fills a "long felt want." W. L. D.

---

HERR PROF. C. WEDL, Professor of Microscopical Anatomy in the University of Vienna, was lately for a brief time a visitor in Chicago, where he was entertained by Prof. DeLaskie Miller, and a few other medical gentlemen.

